# Expectations do not need to be accurate to be maintained: Valence and need for cognitive closure predict expectation update vs. persistence

**DOI:** 10.3389/fpsyg.2023.1127328

**Published:** 2023-02-10

**Authors:** Larissa Henss, Martin Pinquart

**Affiliations:** Department of Psychology, Philipps University, Marburg, Germany

**Keywords:** ViolEx model, coping, expectation, expectation violation, prediction error, need for cognitive closure, valence, achievement

## Abstract

Expectations about us and our environment serve to successfully anticipate the future, make accurate predictions, and guide behavior and decisions. However, when expectations are not accurate, individuals need to resolve or minimize incongruence. Coping is especially important when expectations affect important domains such as students’ academic self-concept. Whether expectations are adjusted after expectation violation (accommodation), maintained by denying the discrepancy (immunization), or whether individuals modify behavior to minimize the likelihood of future expectation violations (assimilation) depends on situational and dispositional predictors. In our experiment, we examined valence of expectation violation (positive vs. negative) as a situational predictor together with need for cognitive closure (NCC) as a dispositional predictor with *N* = 297 participants in a word riddle study. MANCOVA revealed that students tended to assimilate and accommodate more strongly after worse-than-expected achievement, and also NCC promoted both stronger accommodation and assimilation. NCC interacted with the valence of expectation violation: individuals with high NCC reported more assimilation and accommodation only after worse-than-expected achievement. The results replicate and extend previous findings: individuals do not always strive to have the most accurate expectations possible. Instead, both affective (valence) and cognitive (NCC) predictors appear to affect which coping strategy is preferred by the individual.

## Introduction

Expectations can be described as subjective probability distributions of potential situational outcomes (Panitz et al., 2021). Their future-orientation impacts present and future well-being (Rief and Glombiewski, 2017) and determines future behavior (Pinquart and Block, 2020). For instance, educational expectations characterize what individuals realistically expect to achieve (Pinquart and Ebeling, 2020) and these expectations are widely recognized as important predictors of short- and long-term outcomes such as academic achievement and educational attainment (Carolan, 2017). But being future-oriented also implies that expectations are not always accurate: expectations can be confirmed or disconfirmed by future events. Expectation violations are considered to be discrepancies between the prior expectation and the actual outcome (events, experiences, or information; Panitz et al., 2021). Individuals can cope to minimize the impact of expectation violations either by changing/adjusting their expectations or maintaining their expectations despite disconfirming evidence.

The ViolEx (*Viol*ated *Ex*pectations) model postulates different coping strategies: cognitive mechanisms of expectation change (accommodation), cognitive mechanisms to minimize the impact of expectation violations (immunization), and active behaviors to increase the probability of expectation confirmation and decrease the probability of expectation violation (assimilation; Gollwitzer et al., 2018; Panitz et al., 2021). How individuals cope with expectation violations and which strategy is more likely and adaptive is determined by characteristics of the expectation itself as well as situational characteristics and personal dispositions (Panitz et al., 2021; Pinquart et al., 2021). Also regarding educational expectations, coping with expectation violations is likely to differ between situations with better-than-expected outcomes and worse-than-expected outcomes. Thus, the valence of expectation violation can be considered to be a significant predictor of the most likely and adaptive coping strategy. But identical situations may still result in differences in expectation update vs. expectation maintenance because of differences in personal dispositions (Panitz et al., 2021).

Personal dispositions affect and moderate cognitive mechanisms (accommodation, immunization) and active behaviors (assimilation) related to expectation update and expectation maintenance because stable dispositional differences affect the internal representation of the disconfirming situation and of the situational outcome. Furthermore, personal dispositions moderate anticipatory reactions by influencing both ability and motivation to show different behaviors aimed to minimize the impact of the experienced expectation violation. Resulting from this, characteristics of the individual affect the probability of expectation update vs. expectation maintenance in response to expectation violations (Panitz et al., 2021). Expectations can not only be quantified by their valence, but also by the uncertainty that is inherent in them (Panitz et al., 2021). Because individuals differ in their tolerance for uncertainty and ambiguity, the personal disposition need for cognitive closure may affect coping with violated educational expectations.

Based on the assumption that coping is determined by characteristics of the expectation (e.g., educational expectations), situational characteristics (e.g., valence of expectation violation) and personal dispositions (e.g., need for cognitive closure), we aim to investigate differences in coping with better-than-expected vs. worse-than-expected results in an achievement task considering individual differences in need for cognitive closure.

### Characteristic of the expectation: Educational expectations

Coping with violations of educational expectations differs from other expectations, as educational expectations tend to be overly optimistic and particularly resistant to change (Carolan, 2017; Pinquart and Ebeling, 2020; Pinquart and Pietzsch, 2022). Being overly optimistic about one’s abilities and future educational outcomes carries some adaptive consequences that outweigh the inaccuracy of these expectations. Holding positive expectations reduces stress, supports physical and mental health, and increases motivation for exploration and innovation (Sharot and Garrett, 2016). Yet, overly optimistic educational expectations operate in the controversy between these adaptive advantages and disadvantages due to a more frequent need to cope with worse-than-expected outcomes. Therefore, coping with educational expectation violations can be conceptualized as a value-based process to adjust the expectation to embody the highest value (Sharot et al., 2021), without necessarily being a conscious decision (Panitz et al., 2021). Educational expectations tend to be tied closely to an individual’s self-concept (Suárez-Álvarez et al., 2014), which is why the highest accuracy is not always conducive to the most adaptive expectations.

Having optimistic beliefs and expectations about oneself and one’s future has positive effects on the individual’s self-concept (Iovu et al., 2018). If an individual receives expectation-violating information regarding their educational achievement, the advantages of the persistence of the prior expectation are–consciously or unconsciously–compared to the potential advantages of adjusting the expectation. If the advantages of the renewed expectation are perceived to be greater, individuals accommodate and change/adjust the expectation. However, if the integration of the expectation violation into the prior expectation is not seen as beneficial (e.g., because it threatens an individual’s self-concept), individuals immunize against discrepant feedback, and the expectations persists and the self-image remains (Greve and Wentura, 2010) or individuals assimilate and actively change their behavior to avoid future expectation violations. Accommodation tendencies increase when expectation change does not threaten essential parts of a person’s self or their worldview, whereas immunization and assimilation are adaptive to protect self-relevant concepts.

### Characteristic of the situation: Valence of expectation violation

Disconfirming events can be better or worse than expected (Lebois et al., 2016). Contrary to better-than-expected events (positive valence), worse-than-expected events (negative valence) related to educational expectations can threaten individual’s self-concept and individual’s general preference to believe the future is bright rather than dark (Bromberg-Martin and Sharot, 2020). Thus, previous studies found a so-called optimism bias through asymmetric coping depending on the valence of the expectation violation: individuals showed stronger accommodation after better-than-expected events than after worse-than-expected events (Chowdhury et al., 2014; Garrett and Sharot, 2017; Lefebvre et al., 2017; Bromberg-Martin and Sharot, 2020). Adjusting expectations to a lower performance level would potentially be associated with more accurate judgments here, but would also result in the individual suffering from the change in expectation (Sharot et al., 2021). Therefore, individuals often assign more value to expectation persistence and–consciously or unconsciously–cope with immunization or assimilation. Asymmetry is also evident in seeking expectation-confirming information: Individuals are more likely to seek confirmation of positively valued expectations and avoid information seeking that may violate existing positive expectations or confirm negative expectations (Scherer et al., 2012). Even if expectation-violating information is very clear and trustworthy, expectations tend to remain unadjusted if change leads to undesirable outcomes (Eil and Rao, 2011; Kappes and Sharot, 2019). Instead, for affect regulation, individuals preferentially hold on to non-correct expectations associated with positive affect (Sharot and Sunstein, 2020).

The optimism bias is strongly dependent on motivational (motivation to maintain a positive and optimistic view of themselves; Sharot and Garrett, 2016) and emotional factors (feelings predict asymmetric outcomes; Charpentier et al., 2016) and already indicated an asymmetric coping with better-than-expected versus worse-than-expected feedback about educational achievements (Eil and Rao, 2011; Sharot and Garrett, 2016). Nevertheless, there were previous findings with limited evidence for asymmetric coping, because results indicated solely stronger assimilation after experiencing negative rather than positive expectation violations (Henss and Pinquart, 2022), but no differences in accommodation or immunization. Therefore, the influence of valence on coping with expectation violations should be further explored. Individuals seem to consider how coping affects internal states and emotions. A potential threat to the academic self-concept presumably evokes aversive internal states, so negative valence should lead to a stronger tendency toward expectation-persistent strategies. Contrary, positive valence of expectation violation should lead to stronger accommodation because it presumably evokes positive internal states.

### Personal disposition: Need for cognitive closure

Educational expectations are considered to be rather elaborate, certain, and stable constructs with particular significance for an individual’s understanding of the world as they are central elements of the self. Humans rely on their ability to structure information about the world into expectations, schemas, and rules that are simplified models of reality. But individuals differ in their preference for simple or complex models defined as need for cognitive closure (NCC) and therefore in the way they cope with disconfirming information (Webster and Kruglanski, 1994). Higher NCC trait levels should predispose individuals to ignore and resist expectation-inconsistent information (i.e., immunization, assimilation) in order to avoid expectation-discrepant outcomes and protect their models of the world (Neuberg and Newsom, 1993; Schrackmann and Oswald, 2014). This seems to be particularly important for self-relevant characteristics, such as educational expectations. Whereas former studies showed a fairly clear set of findings in which higher NCC was associated with less accommodation or less accommodation-like tendencies (e.g., Dijksterhuis et al., 1996), more recent studies showed strongly context-dependent effects of NCC on coping (Kemmelmeier, 2015; Strojny et al., 2016; Henss and Pinquart, 2022). Higher trait levels may be associated with both stronger assimilation and stronger accommodation because both strategies may reduce uncertainty under some conditions. People are both motivated to hold accurate beliefs and to adapt to changing circumstances but also to defend previously held beliefs and expectations (Schrackmann and Oswald, 2014). Therefore, individuals may sometimes prefer disconfirming information if it is diagnostically more relevant or has a higher utility compared with expectation-confirming information (Schrackmann and Oswald, 2014). On the other hand, individuals may actively search to confirm their beliefs (assimilation) or devalue discrepant information (immunization; Schrackmann and Oswald, 2014) if expectation maintenance is more advantageous (e.g., to academic self-concept) than potentially increasing the accuracy of the expectation.

### The present study

Overall, this study relies on the assumption that coping processes after expectation violations are not only related to external outcomes, but also to internal states. The respective coping with violated educational expectations does not have to be aimed at producing the most accurate expectations possible, but can also be taken in favor of one’s own states and affects. Individuals evaluate the expected consequence of new information, often even unconsciously, and consider how this new knowledge will influence their psychological well-being when integrating it into their educational expectations. Resulting from this, the likelihood of each coping strategy is determined by costs (e.g., more uncertainty, acceptance of unpleasant self-relevant truths) and benefits (e.g., more accurate expectations). In addition to the situational aspect of valence of expectation violation, it should be noted that individuals have a dispositionally stable tendency as to how much they are willing to search for new information and, if necessary, to integrate it into existing concepts. These cognitive processes, as in NCC, may serve as an explanation for why individuals often do not change educational expectations despite disconfirming evidence (Kappes and Sharot, 2019). Accommodation is more likely when expectation change does not threaten people’s self-concept or the essentials of their view of the world (Kahan, 2017). In Sharot and Sunstein’s model (2020), it is evident that both valence with the associated affects and emotions (will the information induce positive or negative feelings, or have no influence on my affect?) and NCC with the associated cognitive processes (will information improve the ability to comprehend and anticipate my reality?) are crucial in coping with expectation violations (Sharot and Sunstein, 2020). Therefore, it seems reasonable that both predictors interact with each other in addition to their main effect on coping with expectation violations. Both predictors revealed significant but partly surprising results in a previous study (Henss and Pinquart, 2022) and will therefore be further explored in a similar study design. The following research questions were addressed: First, does a negative valence of expectation violation lead to stronger assimilation (similar to our previous study; Henss and Pinquart, 2022) as well as stronger immunization and weaker accommodation compared to positive valence of expectation violation? Second, does higher NCC predict stronger accommodation and assimilation (similar to the study by Henss and Pinquart, 2022) and stronger immunization (as found by Schrackmann and Oswald, 2014)? Third, do valence of expectation violation and NCC interact in predicting accommodation, assimilation, and immunization? Our study aims to shed light on the way in which the situational characteristic valence and the personal disposition NCC explain differences in coping after expectation violations.

## Methods

The study was conducted online via SoSci Survey in a 45-min experiment. The study was approved in advance by the local ethics committee of the department of the researchers (reference number 2022-16 k). In addition, all participants confirmed written informed consent and were treated according to the ethical guidelines of the German Society of Psychology and the Declaration of Helsinki. We report how we determined our sample size, all data exclusions (if any), all manipulations, and all measures in the study.

### Sample and participants

Sample size planning via G*Power resulted in a required number of participants of *n* = 278 (MANOVA: Global Effects, calculated effect size *f*^2^ = 0.04, alpha = 0.05, *ß* = 0.80, number of groups: 2, response variables: 3), based on the effect sizes in a comparable study by Henss and Pinquart (2022). Because of expected exclusions, we stopped recruitment after *n* = 297 participants completed the questionnaire. Participants were recruited via email distribution lists from different German universities. Inclusion criteria were a minimum age of 18 years, very good German language skills, and registration at a university. To compensate for their efforts, the participants could take part in a raffle for two 50€ value vouchers.

### Randomization and procedure

The study was conducted online with computer-based randomization to manipulate the valence of expectation violation as a one-factor between-subjects design. The personality variable NCC was assessed as a covariate and the coping strategies accommodation, assimilation, and immunization as dependent variables. Data collection took place from May to July 2022.

The study was advertised with a cover story as an investigation of students’ linguistic abilities in word riddles. The supposed aim of the study was to determine whether the linguistic abilities of academics differ significantly from those of non-academics either at the stage of graduation or at the stage of post-graduate professional practice. The participants were told that their linguistic abilities were determined by their performance in an anagram test. Participants’ demographic data and NCC were collected before participants were given an introduction to their task. To manipulate and induce an expectation violation, anagrams were used in which randomly arranged letters must be rearranged to form a word. Anagrams have been used as an effective means of violating performance expectations in a variety of previous studies (e.g., Boyes and French, 2010; Koppe and Rothermund, 2017). After introduction to the task, all participants completed an identical training task. Participants were told that they would complete a total of four runs of 11 anagrams each. In fact, only three runs were completed, but the misinformation was unavoidable to allow for a valid assessment of coping strategies after the third run.

### Experimental manipulation

To manipulate the valence of expectation violation, we systematically varied between the positive versus negative valence of expectation violation at three points: test-taking information, anagram solvability, and standardized performance feedback differed between the positive and negative valence groups, similar to the successful manipulation of valence in a previous study (Henss and Pinquart, 2022).

#### Pre-trial information at test onset

Participants received information about the performance of previous trial test persons in accordance with their condition. In the negative valence group, in which positive expectations were to be established, participants were told that previous participants were able to solve a relatively high number of anagrams per trial (on average 9 out of 11). In the positive valence group, on the other hand, in which negative expectations were to be established first, the participants were told that a low number of anagrams could previously be solved per run (on average 4 of 11). Subsequently, participants were asked for the first time about their performance expectations for the next run.

#### Pre-expectation violation–Solvability of anagrams and performance feedback

The anagrams varied in difficulty between the positive valence group and the negative valence group in order to stabilize participants’ expectation. In the positive valence group, where low expectations were initially to be generated, participants were given four easily solvable and seven unsolvable anagrams so that the negative performance feedback (“You could not solve more than 4 of the 11 anagrams correctly”) was valid. In the negative valence group, where high expectations were to be generated, participants received 11 easily solvable anagrams and feedback that they had performed above average (“you were able to solve at least 9 of the 11 anagrams correctly”). The second run was structured identically. Participants were given new anagrams, but they met the criteria of the first run. Performance feedback was also identical to the previous run.

#### Expectation violation

According to the assignment to the positive or negative valence group, an expectation violation was induced in the third and last run. In the positive valence group, after building up low expectations, participants now received 11 easily solvable anagrams and feedback that their performance exceeded their expectations. In the negative valence group, after building high expectations, participants now received four easily solvable anagrams and seven unsolvable anagrams, along with feedback that their performance was below their expectations. Finally, the subjects’ coping strategies were assessed, with the cover story that we wanted to elicit reasons for the discrepancy with the previous runs. Subsequently, the study ended and participants were informed of the true intention of the study.

### Measures

#### Socio-demographics

At the beginning of the study, we assessed age, gender, and field of study, as well as current semester of study and type of study (bachelor vs. master). The information was collected to increase the credibility of the cover story on the relationship between test performance and level of academic education.

#### Manipulation check

To check for the generation of positive and negative expectations, we assessed how many anagrams participants expected to solve before each run. This allowed us to check if participants actually experienced an expectation violation. Participants could state performance expectations between 0 and 11 anagrams per run.

#### Need for cognitive closure

The personality trait NCC was assessed prior to the start of the test runs using Schlink and Walther’s (2007) 16NCCS (Need for Cognitive Closure Scale). The scale consists of 16 items that were answered on a 7-point scale ranging from 1 = strongly disagree to 7 = strongly agree. Cronbachs alpha indicated good internal consistency with α = 0.84.

#### Coping strategies

To capture coping strategies, we relied on scales previously used in a similar study (Henss and Pinquart, 2022). This scale was revised and adapted to the anagram paradigm. The revision was mainly related to the immunization subscale in order to increase its internal consistency by focusing more on the subfacets denial and devaluation. The final scale consisted of 22 items and a 7-point Likert scale ranging from 1 = strongly disagree to 7 = strongly agree. Accommodation was assessed with 6 items (e.g., “In the future, I will try to be more realistic about my performance.”) and had good internal consistency with α = 0.86. Assimilation was assessed with 7 items (e.g., “I will pay more attention in the next test block to make sure that my previous expectation comes true.”) and had acceptable internal consistency with α = 0.75. Immunization was assessed with nine items (e.g., “The performance in the last run was atypical for me.”) and had acceptable internal consistency with α = 0.74.

### Data analysis

To calculate main statistical effects of the manipulated variable valence and the personality trait NCC, as well as their interaction with respect to the three coping strategies, a MANCOVA was performed for the overall model with valence as an independent variable and NCC as covariate as well as their interaction. The MANCOVA was calculated at a significance level of 5%.

#### Transparency and openness

We report how we determined our sample size, all data exclusions, all manipulations, and all measures in the study. All data and research materials are available at Open Science Framework (DOI 10.17605/OSF.IO/EBUJM). Data were analyzed using IBM SPSS Statistics 29. Our study design and its analysis were not pre-registered.

## Results

### Participants

We checked data of all *n* = 297 participants for univariate outliers via Box-Whisker plots and for multivariate outliers via Mahalanobis Distance (*p* < 0.001). Whereas univariate outliers were checked for plausibility and if necessary, only excluded pairwise, we excluded three data sets from further analysis because they were identified as multivariate outliers. Furthermore, 26 data sets of participants were excluded because the participants had either detected the experimental manipulation or had not experienced an expectation violation. The former was indicated qualitatively by a free response field at the end of the study, where participants could indicate what they thought the background of the study was. Here, exclusion occurred when subjects indicated that the study goal was to analyze coping with expectation violations and additionally indicated that they did not find the manipulation credible (e.g., „I assume that the feedback was not related to my actual performance, but to investigate coping with violated expectations“). The second was indicated quantitatively by specifying that participants in the negative valence group had the expectation of solving at least six anagrams and that participants in the positive valence group had the expectation of solving a maximum of six anagrams.

The final sample consisted of *n* = 268 participants. The participants were mainly young adults (*M* = 23.87, S*D* = 4.98), female (*n_female_* = 215, *n_male_* = 73, *n_diverse_* = 9), bachelor students (*n_bachelor_* = 204,*n_master_* = 93) in the fields of psychology (*n* = 108), social sciences (*n* = 83), natural sciences (*n* = 63), teaching (*n* = 22) or others (*n* = 25).

### Manipulation check

To verify that expectations differed between groups, we compared the reported expectations before the manipulation of the valence of expectation violation. There should be significantly lower expectations in the positive valence group compared with the negative valence group. Independent *t*-tests revealed that participants’ expectations differed significantly between the groups (*t_1_*(266) = −10.54, *p* < 0.001; *t_2_*(215) = − 25.14, *p* < 0.001; *t_3_*(266) = − 35.14, *p* < 0.001) and that the difference between the groups increased with each run (*M_1p_* = 5.31, *SD_1p_* = 1.79 vs. *M_1n_* = 7.66, *SD_1n_* = 1.86; *M_2p_* = 3.72, *SD_2p_* = 1.14 vs. *M_2n_* = 8.34, *SD_2n_* = 1.78; *M_3p_* = 3.30, *SD_3p_* = 1.25 vs. *M_3n_* = 8.88, *SD_3n_* = 1.35). It can be concluded that the manipulation of the valence of expectation violation was successful, and significantly lower expectations were induced in the positive valence group compared with the negative valence group. Expectations solidified over time and, on average, corresponded in the run before the expectation violation to the expectation values that participants received in advance as information about prior performance.

### MANCOVA

**Figure 1:**
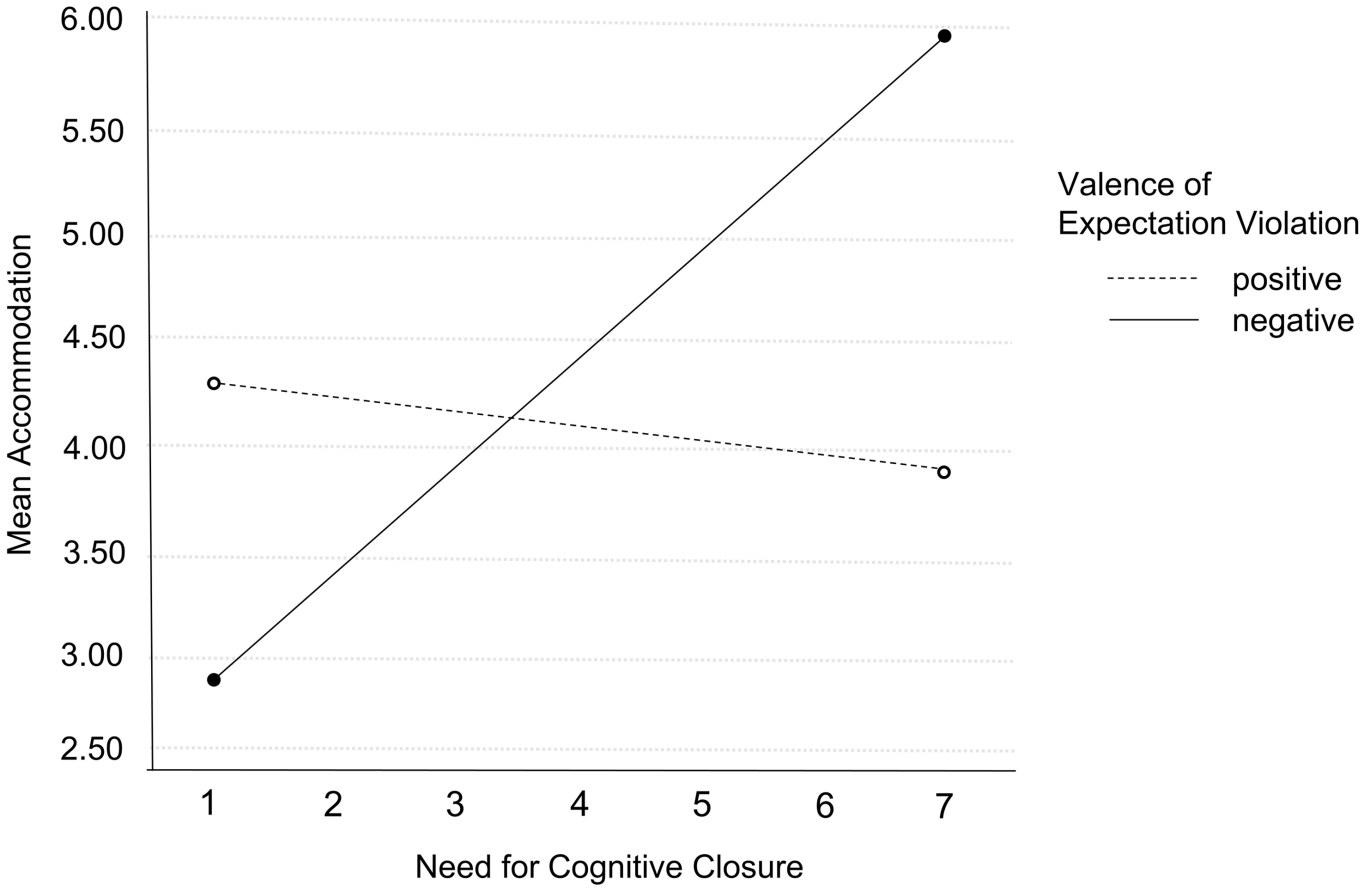
Interaction effect of valence of expectation violation and NCC on accommodation. All variables were measured on a 7-point Likert scale.

To evaluate both the main effects of NCC and valence of expectation violation and their interaction effect regarding the dependent variables accommodation, assimilation, and immunization, we conducted a MANCOVA. The main effects analysis showed statistical significance for both valence and NCC. Valence of expectation violation was a significant predictor in the overall model (Wilk’s *Λ* = 0.96, *F*(3, 262) = 3.26, *p* = 0.02, *η_p_^2^* = 0.03) and revealed a significant effect on accommodation (*F*(1, 264) = 9.66, *p* < 0.01, *η_p_^2^* = 0.04) and assimilation (*F*(1, 264) = 4.19, *p* = 0.04, *η_p_^2^* = 0.02). Positive valence of expectation violation was associated with less accommodation (*M_p_* = 4.09, *SD_p_* = 1.09 vs. *M_n_* = 4.24, *SD_n_* = 1.10), and negative valence with stronger assimilation (*M_p_* = 3.95, *SD_p_* = 0.93 vs. *M_n_* = 4.23, *SD_n_* = 0.88). However, there was no significant effect of valence of expectation violation on immunization (*F*(1, 264) = 0.10, *p* = 0.77; *M_p_* = 4.28, *SD_p_* = 0.94 vs. *M_n_* = 4.69, *SD_n_* = 0.84). The covariate NCC was also a significant predictor in the overall model (Wilk’s *Λ* = 0.97, *F*(3,262) = 2.99, *p* = 0.03, *η_p_^2^* = 0.03). Here, statistically significant effects were found on accommodation (*F*(1, 264) = 6.99, *p* < 0.01, *η_p_^2^* = 0.03) and assimilation (*F*(1) = 6.37, *p* = 0.01, *η_p_^2^* = 0.02), but not on immunization (*F*(1, 264) = 0.07, *p* = 0.79, *η_p_^2^* = 0.00). Individuals with higher NCC reported stronger accommodation *and* assimilation. As well, the interaction of the two independent variables valence and NCC was significant (*F*(3, 262) = 4.50, *p* < 0.01, *η_p_^2^* = 0.05) and showed a statistical effect on accommodation (*F*(1, 264) = 11.76, *p* < 0.01, *η_p_^2^* = 0.04) and assimilation (*F*(1, 264) = 6.85, *p* < 0.01, *η_p_^2^* = 0.03), but again not on immunization (*F*(1, 264) = 0.17, *p* = 0.68, *η_p_^2^* = 0.00). Whereas higher NCC was associated with slightly less accommodation in the positive valence group, the opposite was true in the negative valence group: the higher a participant’s NCC, the more accommodation was reported (see Figure 1).

**Figure 2:**
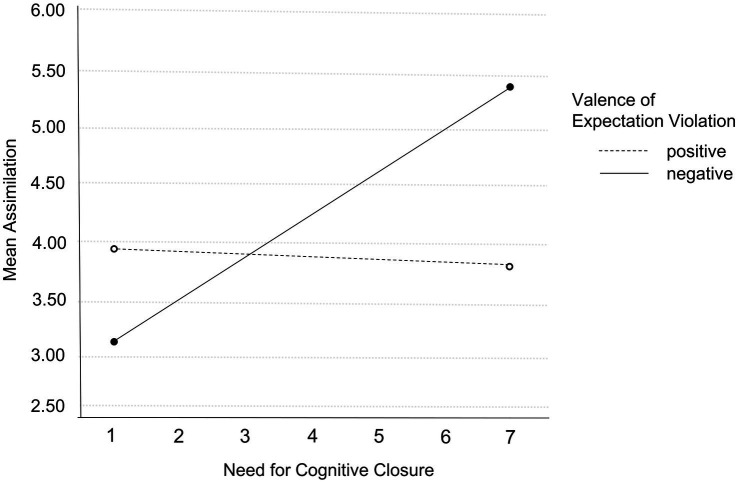
Interaction effect of valence of expectation violation and NCC on assimilation. All variables were measured on a 7-point Likert scale.

With regard to assimilation, the same pattern emerged: in the positive valence group, participants with higher NCC reported slightly less assimilation, whereas in the negative valence group, with increasing NCC, more assimilation was reported (see Figure 2). Overall, analysis of the total MANCOVA model revealed medium effect sizes for accommodation (Wilk’s *Λ* = 0.95, *F*(3, 264) = 5.84, *p* = 0.001, *η_p_^2^* = 0.06) and assimilation (*F*(3, 264) = 4.76, *p* = 0.001, *η_p_^2^* = 0.06) and small effect size for immunization (*F*(3, 264) = 4.67, *p* < 0.01, η_p_^2^ = 0.05) according to Cohen’s taxonomy (Cohen, 1988).

## Discussion

The aim of the study was to investigate both situational characteristics in the form of valence of expectation violation and personal dispositions with NCC as predictors of coping with expectation violation in the context of educational expectations. Based on the ViolEx model, our aim was to investigate how both predictors affect cognitive and behavioral coping strategies after expectation violations. In accordance with Sharot and Sunstein’s model (2020), affective and emotional aspects determined by valence as well as cognitive mechanisms determined by NCC were included (Sharot and Sunstein, 2020). We identified valence and NCC as significant predictors of coping with expectation violations and obtained comparable, partly surprising results similar to those of the previous study (Henss and Pinquart, 2022). Whereas positive valence led to less accommodation, negative valence led to more assimilation. Stronger NCC again led to both more assimilation and accommodation, but was not related to immunization. The interaction of both predictors showed that the effect of NCC on coping strategies was valence-dependent: the significant effect on assimilation and accommodation was evident only after negative valence of expectation violation.

### Characteristic of the situation: Valence

Educational expectations tend to be overly optimistic and do not necessarily need to be accurate to be adaptive (Garrett et al., 2018). Expectation violations might be costly in certain situations, but advantageous in others when their benefits outweigh their costs (McKay and Dennett, 2009). This cost–benefit trade-off should be particularly important when considering the valence of the expectation violation and should lead to differences in coping: former studies indicated an optimism bias, because individuals integrated information into expectations asymmetrically based on the valence and therefore desirability of the information. We suspected that especially for self-relevant beliefs like educational expectations, individuals protect their academic self-concept through coping related to expectation maintenance after negative valence of expectation violation (Greve and Wentura, 2010) and positively adjust their academic self-concept through expectation update after positive valence.

Our first finding was not in line with an optimism bias: individuals reported less accommodation after an expectation violation with positive valence. This could possibly be related to the measurement of accommodation, which states, among others, that the respondent will try to form more realistic expectations in the future. However, individuals might have been encouraged by the positive feedback to be more optimistic, which would also be consistent with the theoretical approach of overly optimistic educational expectations.

Nevertheless, our second finding was in line with asymmetric coping after expectation violation and the result of our former study: individuals reported more assimilation after an expectation violation with negative valence. Thus, when individuals are confronted with a reality in which their positive achievement expectations are not met, they report active behavioral tendencies aimed at fulfilling their future expectations despite worse-than-expected present feedback. Increasing effort to compensate for a worse-than-expected performance can be considered as adaptive and in line with theoretical assumptions of self-concept protection and stability of educational expectations. Expectation update is more likely for uncertain expectations and less likely for certain and elaborated expectations such as educational expectations in university (Spicer et al., 2020).

It is nevertheless surprising that, after worse-than-expected feedback, individuals reported stronger assimilation, but not stronger immunization, which is also considered to be an expectation-maintaining strategy for self-concept protection (Greve and Wentura, 2010). The lack of significance could have both theoretical and methodological reasons: First, the dependence of the effect of negative valence on immunization on a high degree of expectation violation found in a previous study could explain the absence of the effect in this study (Henss and Pinquart, 2022). If the feedback in our study was not perceived as “expectation-violating enough” because we did not include information about a high degree of expectation violation, participants probably had no incentive to immunize. Performance feedback could be adjusted by clearly indicating that performance was strongly above or below expectations. Second, it should be noted that immunization as an unconscious and non-intentional process is difficult to capture by an explicit self-report measure. Recent literature suggests that immunization as an automatic process might be adequately assessed via indirect measures (Rief et al., 2022) or that the different facets of immunization might be captured via open questions and qualitative analysis (Kube et al., 2022).

### Personal disposition: Need for cognitive closure

Previous studies have suggested that the effects of NCC on coping are context-dependent and may promote both expectation update and expectation maintenance. Our results support this assumption and are perfectly in line with Sharot and Sunstein’s thoughts (2020): information can enhance or reduce individual’s view to comprehend their environment and disconfirming information challenges people’s existing models and schemata (Sharot and Sunstein, 2020). In the present study, simply ignoring the discrepancy between expected and actual achievement would not be the best way of coping for individuals with high NCC as they believed to participate in a fourth run immediately thereafter which could provide additional expectation violations. Therefore, immunization did not differ between individuals with higher vs. lower NCC. Individuals strive to make accurate predictions and therefore integrated performance feedback through expectation change or behavior adjustment to ensure that expectations are less likely to be violated in the future. Thus, adjustment of expectation in the direction of the formerly unexpected feedback (accommodation) or active behavior of creating a reality that conforms to prior expectations (assimilation) are more likely to fulfill their need for clarity and structure, and to the avoidance of uncertainty compared with denial or devaluation (immunization).

Accommodation is related to the improvement of existing expectations, schemata and models with new information to improve the fit between expectation and reality (Sharot and Sunstein, 2020). Stronger accommodation after disconfirming information leads to more comprehension of the world and, therefore, fulfills the needs of an individual with high NCC.

Assimilation is related to the active avoidance of information that is suspected to weaken the understanding of the world. Stronger assimilation after disconfirming information promotes a fit between the internal representation of the expectation and reality by actively changing the reality of which individuals are aware (Sharot and Sunstein, 2020).

Interestingly, in our study, accommodation and assimilation are not mutually exclusive, but positively correlated with each other (see also Henss and Pinquart, 2022). According to the ViolEx model, it is assumed that expectation violations may lead to accommodation (which can be both expectation update but also “only” expectation destabilization), which can in turn motivate stronger assimilation to restore confidence. Moreover, the combination of modest expectation adjustment and efforts to meet expectations may potentially be an adaptive approach to minimize the magnitude of future expectation violations. Individuals might accept a new reality, but nevertheless strive to meet the prior expectation. This conclusion seems especially plausible when considering the interaction of both predictors in the following paragraph.

### Valence and NCC

As effects of NCC seem to be context-dependent and may be determined by the assigned value or advantage of beliefs, effects of NCC likely depend on the valence of expectation violation (i.e., will accepting a new reality be advantageous or will maintaining existing schemata despite less accuracy be advantageous?). The evaluation of information is a non-intentional, unconscious cognitive process and strongly sensitive to motivational influences like valence of the expectation violation (Schrackmann and Oswald, 2014). Indeed, there were differences between positive and negative valence: stronger accommodation and assimilation of individuals with high NCC were only found after experiencing a negative valence of expectation violation. No difference was found for positive valence, and this pattern was shown for both accommodation and assimilation. NCC seems to depend on valence, and the trait NCC seems to be particularly important for coping with expectation violations when individuals experience a worse-than-expected reality. Possibly, a negative violation of expectations causes a stronger need for regulation due to stress, which in turn could lead to a stronger impact of personality dispositions like NCC on behavior.

## Limitations and conclusions

As in previous studies on the ViolEx model, the internal consistencies of some scales on coping with expectation violations were less than optimal in this study. The ViolEx model is still a very new theoretical model that has only been empirically researched in recent years, and experimental research in particular is still in its infancy. Therefore, there is still a need for optimization with regard to the measurement of coping processes. But it should be noted that independently of the ViolEx model, the measurement of immunization has so far proven to be very difficult (Brandtstädter, 2007).

It should also be noted that the study was conducted with feedback on achievement expectations which are strongly shaped by prior experiences and expectations (Andrew and Hauser, 2011; Carolan, 2017). Therefore, the generated expectation must always be considered in the context of generalized expectations and other cognitions. A certain and elaborated expectation that has often been confirmed in the past would less likely change as a result of a single expectation violation than an expectation that is associated with less prior experience or has been disconfirmed more frequently. For future studies, it might be beneficial to integrate general educational expectations independent from the achievement task itself, because coping might be biased by previous experiences and expectations. Furthermore, this information can be used to differentiate between individuals who base their self-esteem more strongly on achievement expectations than others in order to investigate if immunization processes are more strongly reported in individuals with a potentially higher threat to their academic self-concept (Greve and Wentura, 2003).

In our study, we replicated and expanded our former results that coping with expectation violation in an achievement context is predicted by situational characteristics and personal dispositions. The context-dependent effects of NCC are partly based on valence, because higher NCC seems to be of high relevance when facing a worse-than-expected reality, but not when facing a better-than-expected reality. Higher NCC again resulted in stronger accommodation and assimilation, indicating that both coping strategies seem to be not mutually exclusive coping strategies in this context, although they seem to be contradictory by definition. Finally, our results indicate that expectations do not always need to be accurate to be adaptive — individuals are sometimes reluctant to update their expectations because it would evoke negative feelings and therefore even actively adjust their behavior to confirm and protect prior expectations. Provided that events can be influenced and controlled to some extent, active behavioral change through assimilation is the most adaptive strategy to respond to events with negative valence. Assimilation can reduce the likelihood of future disappointment and negative affect and furthermore avoids the negative feelings associated with lowering expectations through accommodation.

## Data availability statement

The datasets presented in this study can be found in online repositories. The names of the repository/repositories and accession number(s) can be found at: https://osf.io/ebujm/ (DOI 10.17605/OSF.IO/EBUJM).

## Ethics statement

The studies involving human participants were reviewed and approved by Philipps University Marburg, Ethics Committee in Psychology (FB04). The patients/participants provided their written informed consent to participate in this study.

## Author contributions

LH was the main contributor to the conception and design of the study, and organized the database and performed the statistical analysis. LH wrote the manuscript and MP added his ideas and corrections several times. LH and MP contributed to manuscript revision, read, and approved the submitted version.

## Funding

This work was funded by the Deutsche Forschungsgemeinschaft (DFG, German Research Foundation) – project number 290878970-GRK 2271, project 7. Open Access funding provided by the Open Access Publishing Fund of Philipps-Universität Marburg with support of the Deutsche Forschungsgemeinschaft (DFG, German Research Foundation).

## Conflict of interest

The authors declare that the research was conducted in the absence of any commercial or financial relationships that could be construed as a potential conflict of interest.

## Publisher’s note

All claims expressed in this article are solely those of the authors and do not necessarily represent those of their affiliated organizations, or those of the publisher, the editors and the reviewers. Any product that may be evaluated in this article, or claim that may be made by its manufacturer, is not guaranteed or endorsed by the publisher.
